# Compressive strength evaluation of thin occlusal veneers from different CAD/CAM materials, before and after acidic saliva exposure

**DOI:** 10.1007/s10266-022-00741-5

**Published:** 2022-09-12

**Authors:** Codruța Ille, Elena-Alina Moacă, Daniel Pop, Luciana Goguță, Carmen Opriș, Ioana Ligia Pîrvulescu, Liane Avram, Andrei Faur, Anca Jivănescu

**Affiliations:** 1 Department of Prosthodontics, Faculty of Dental Medicine, “Victor Babes” University of Medicine and Pharmacy, Revolutiei Ave. 1989, No. 9, 300580 Timișoara, Romania; 2 TADERP Research Center—Advanced and Digital Techniques for Endodontic, Restorative and Prosthetic Treatment, “Victor Babeș” University of Medicine and Pharmacy, Revolutiei Ave. 1989, No. 9, 300041 Timişoara, Romania; 3 Department of Toxicology and Drug Industry, Faculty of Pharmacy, “Victor Babeș” University of Medicine and Pharmacy Timisoara, 2nd Eftimie Murgu Square, 300041 Timisoara, Romania; 4 Research Centre for Pharmaco-Toxicological Evaluation, “Victor Babeș” University of Medicine and Pharmacy, 2nd Eftimie Murgu Square, 300041 Timișoara, Romania; 5 Department for Materials and Manufacturing Engineering, Faculty of Mechanics, Politechnic University of Timisoara, Mihai Viteazu Ave., No. 1, 300222 Timisoara, Romania

**Keywords:** Occlusal veneers, Compressive strength, Tooth wear, CAD/CAM materials, Artificial saliva storage

## Abstract

In the present study are depicted valuable observations for practitioners, obtained from an in vitro study which aims to evaluate the compressive strength of occlusal veneers fabricated from 3 type of restorative materials, before and after 1 month of acidic artificial saliva exposure (pH = 2.939). In this context, 90 extracted human molars were prepared to receive computer-aided design/computer-aided manufacturing (CAD/CAM) occlusal veneers. The restorative materials considered in this study were: Cerasmart; Straumann Nice and Tetric CAD. The occlusal veneers were designed, milled and cemented with an adhesive dual-cure resin cement. From all the extracted human molars, only sixty specimens were immersed in acidic artificial saliva, for 1 month, at 37 °C ± 1 °C and part of this specimens were also thermo-cycled, between 5 and 55 °C ± 2 °C, before compressive strength test. The results showed a lower compressive strength for both the samples exposed to acidic artificial saliva as well as for the samples exposed to acidic artificial saliva and thermo-cycled. Scanning electron microscopy (SEM) showed that after compressive strength, all the specimens non-exposed to acidic artificial saliva, present extensive cracks formation at the surface of the restorations, and after exposure to acidic artificial saliva for 1 month, the surface damage was characterized by longitudinal and profound fractures of the restoration, as well as the fracture of the tooth structure. Between CAD/CAM materials tested, nanoceramic resin shows more favorable fracture patterns, both before and after acidic artificial saliva exposure.

## Introduction

Restorative dentistry emphasizes with the preservation of dental structures. An ideal dental restoration should present perfect marginal adaptation, biocompatibility with oral environment, esthetics and long-term mechanical strength [1, 2]. Severe tooth wear is defined as a significant loss of tooth structure with dentin exposure and important loss of the clinical crown. The last decade showed an increase in the prevalence of dental wear, especially in young people. Minimally invasive treatment approach has become an important alternative to traditional tooth preparation. Recent advances regarding the CAD/CAM materials and technology were made, offering new possibilities for the restorations of serious worn dentition, where space is narrow [3]. The development of new adhesive materials and techniques create the possibility to restore tooth wear with thin occlusal veneers, milled from different CAD/CAM blocks. To take full advantage of the benefits of these materials, new research regarding mechanical strength is useful [4, 5]. Knowledge regarding the mechanical properties of a restorative materials has a great significance to researchers and clinicians, because the extensive fracture of these materials has been reported as the major cause of failure [6]. The mechanical strength of a restorative material largely depends on its composition, but endogenous and/or exogenous factors (i.e., acidic beverages, gastric acid, water sorption, cariogenic biofilm or salivary enzymes) may also affect the mechanical strength, by material degradation [7–9]. Endogenous acids degrade both dental structure and restoration, due to the low pH value [10]. Gastric juice is an endogenous acid with a pH ranging from 1.0 to 3.0, being highly present in the oral environment especially at patients with gastroesophageal reflux or other related disorder [11]. For this type of patients, dental rehabilitation must be taken much more seriously and can be done by direct or indirect restoration depending on the severity of the disorder. Due to the esthetics and durability limitations, the direct restoration technique of tooth wear using composite material is not an optimal treatment modality over time [12]. Contrary, the ceramic materials do not show the limitations above, but the feasibility of using a thin surface depends on the fabrication techniques and compressive strength. Ceramic hybrid materials are considered a new generation of indirect restoration, due to the fact that they are composed of inorganic ceramic fillers and poly-functional methacrylate monomers [13].

The CAD/CAM restorative materials allows a precise reproduction of the tooth preparation design (veneers, full contour and partial restorations even fixed restorations with 3 elements) [14, 15], becoming more popular due to the numerous advantages, namely: lower costs, time-saving, stable quality of materials and improved physical, chemical, and mechanical properties [16, 17].

The newest composites as well as ceramic hybrid materials allows milling surface even at a thin thickness to conserve the remaining substance of tooth. Occlusal veneers (table tops) from composite resin blocks have higher fatigue resistance than reinforced ceramics [16]. The occlusal veneers are extra-coronal restorations, used to protect tooth structure, which require simple treatments modalities, based on anatomical considerations and interocclusal clearance. In addition, the occlusal veneers are easy to manufacture and highly useful in advanced erosion and attrition. Minimal tooth preparation is recommended to remove the superficial aprismatic enamel that gives low bonding strength to the composite resin. It also requires careful preparation to avoid exposing the dentine which would result in lower bonding strength. Enamel thickness is assumed to range from 0.4 to 0.7 mm. Most of the researchers recommend 0.5 mm thickness for porcelain laminate veneer [18].

CAD/CAM composite materials, combine the essential properties of strength and elasticity to reduce the tooth preparation and produce a better marginal fit [17]. Recent studies reported that resin-based ceramic materials possess good mechanical features [19], with surface properties quite analogues to natural teeth [20]. Between the advantages of hybrid ceramic materials are both chairside fabrication without subsequent steps as well as esthetics and mechanical properties similar with those of lithium disilicate [21]. Moreover, another important advantage for dental restorations is the full digital workflow, from intraoral scanning, to the digital design and milling of various CAD/CAM materials [22, 23].

The outcomes of several studies regarding the partial coverage of ceramic restorations, are contradictory. Even though most producers recommend posterior ceramic restoration with a thickness of minimum 1.5 mm, other research studies considered ceramic restorations with a thickness of 1.0 mm, even smaller with satisfactory long-term clinical results [14–16, 24–26].

Factors such as humidity, pH or temperature from oral cavity led to conversion of dental material characteristics, as a function of their composition [27]. These factors must be kept under control to prevent the slow propagation of cracks, caused by corrosion, moisture-assisted or low pH in the oral environment. The appearance and propagation of cracks, lead to mechanical damage, and, therefore, to the resistance reduction over time [28].

In this regard, the purpose of the present study was the assessment of the compressive strength results of occlusal veneers milled from three type of CAD/CAM materials, with an ultrathin thickness of 0.5 mm and the degree of surface damage, before and after exposure for 1 month to acidic artificial saliva. The research hypothesis is that the use of 0.5 mm ultrathin occlusal veneers will be as resistant as thicker occlusal veneers of 0.7–1 mm when applying a higher compressive force as normal masticatory forces. In addition, we suppose that the immersion of the entire tooth-veneer complex in acidic artificial saliva, as well as thermal cycling study contributes to the deterioration of its structure. In this sense, we want to determine the value of the maximum compression force, which can be applied to the whole complex and the degree of damage of the occlusal veneers with ultrathin dimension (0.5 mm).

The outcomes of the present study can be regarded as valuable observations for practitioners when they need to choose the most suitable materials in terms of mechanical properties, for the restoration of dental wear.

## Materials and methods

### Materials

Three different CAD/CAM materials for chair side milling machines were considered in this study: a nanoceramic resin (Cerasmart, GC Europe Dental Products, Tokyo, Japan), a lithium-disilicate-strengthened lithium aluminosilicate glass ceramic (Straumann Nice, Freiburg, Germany) and a composite resin (Tetric CAD, Ivoclar Vivadent, Schaan, Liechtenstein). A complete description of the evaluated CAD/CAM material bocks is presented in Table 1.

**Table 1:** Chemical composition of the materials tested

Material	Classification	Composition	Manufacturer
Cerasmart	Nanoceramic resin	Composite resin material with 71% silica and barium glass nanoparticles	GC Europe Dental Products (Tokyo, Japan); https://www.gcamerica.com
Straumann Nice	Glass ceramic	70% SiO_2_, 11% Li_2_O, 11% Al_2_O_3_, 3% K_2_O, 2% Na_2_O, 8% P_2_O_5_, 0.5% ZrO_2_, 2% CaO, 9% coloring oxides	Nice, Straumann (Freiburg, Germany); https://www.straumann.com
Tetric CAD	Composite resin	Nanohybrid composite resin with barium glass (< 1 mm) and silicon dioxide fillers (< 20 nm)	Ivoclar Vivadent (Schaan, Liechtenstein); https://www.ivoclarvivadent.com

### Specimens preparation

**Fig. 1:**
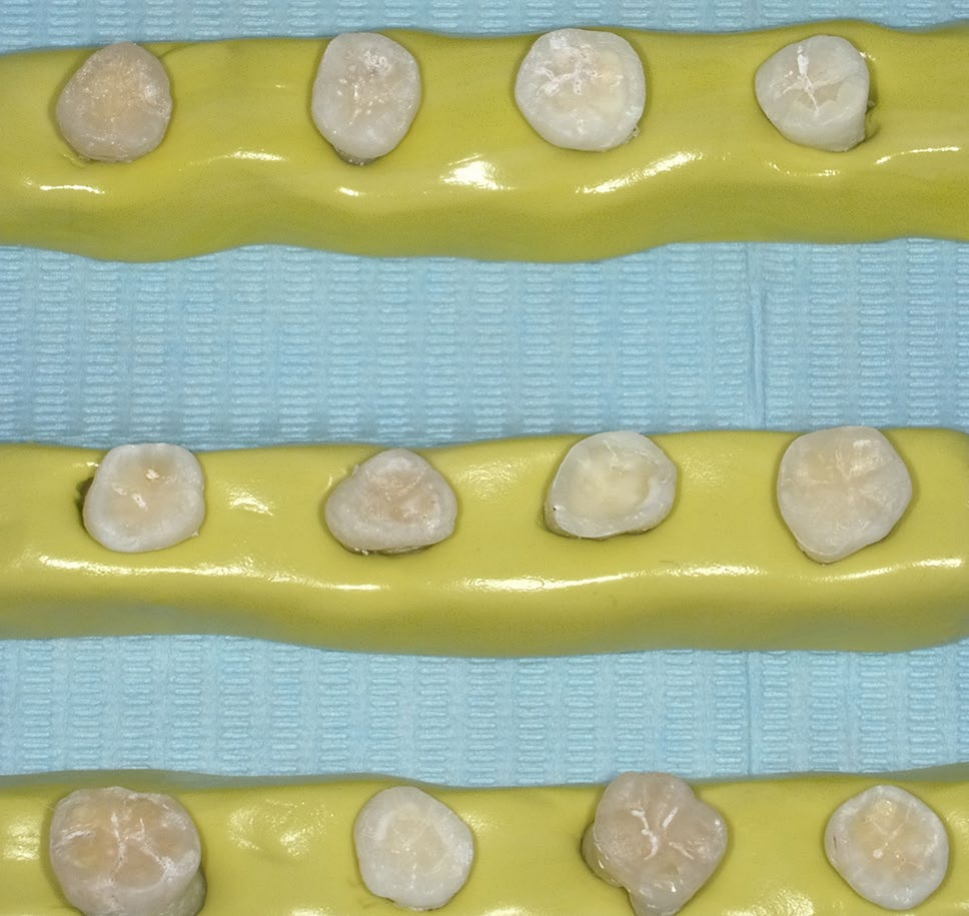
Prepared extracted molars before digital impression and cementation

Ninety intact maxillary human molars with average dimensions of 11.5 ± 2 mm bucco-lingually and 10.0 ± 2 mm mesiodistal crown width, were selected. All subjects gave their informed consent before their molars were extracted. The study was conducted in accordance with the Declaration of Helsinki, and the protocol was approved by the “Victor Babes” University of Medicine and Pharmacy Ethics Committee with the approval number 47/29.09.2020. After extraction procedure, all the specimens were disinfected by immersion in 1% chloramine T solution, for 3 days and then, the soft tissue remnants were removed using a scaler (Goldman, Illinois, USA). For easy handling of the specimens, the human molars were inserted in putty polyvinylsiloxane (Elite HD, Zhermack, Italy). The occlusal enamel of each tooth crown was removed, the fissures were enlarged and the sharp margins were rounded off to simulate tooth wear. All teeth were prepared by the same prosthodontics specialist with identical preparation equipment and finishing set (Komet USA, Rock Hill, SC, USA) (Fig. 1).

Part of the specimens that were not selected to be exposed to acidic artificial saliva (*n* = 30) were stored in physiological serum to avoid desiccation and the saline solution was changed once a week throughout the study. The other remaining part of specimens (*n* = 60) were immersed in acidic artificial saliva, for 1 month, at 37 °C ± 1 °C.

### Scanning procedures and restoration design

**Fig. 2:**
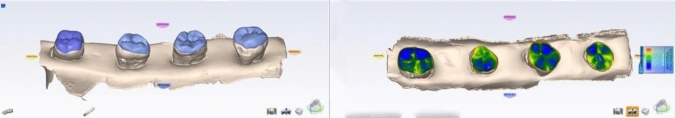
Scanned occlusal veneers digital design

Using the intraoral scanner (PlanScan, Planmeca, Helsinki, Finland), a digital impression of each prepared tooth was taken. Using the PlanCAD^®^ Easy (Planmeca—www.planmeca.com), the restoration design was made. Occlusal veneers were designed with a homogenous thickness of 0.5 mm based on an anatomic tooth, with similar occlusal morphologies, and marginal adaptation of the individual preparations maintained (Fig. 2).

### Occlusal veneers manufacturing

**Fig. 3:**
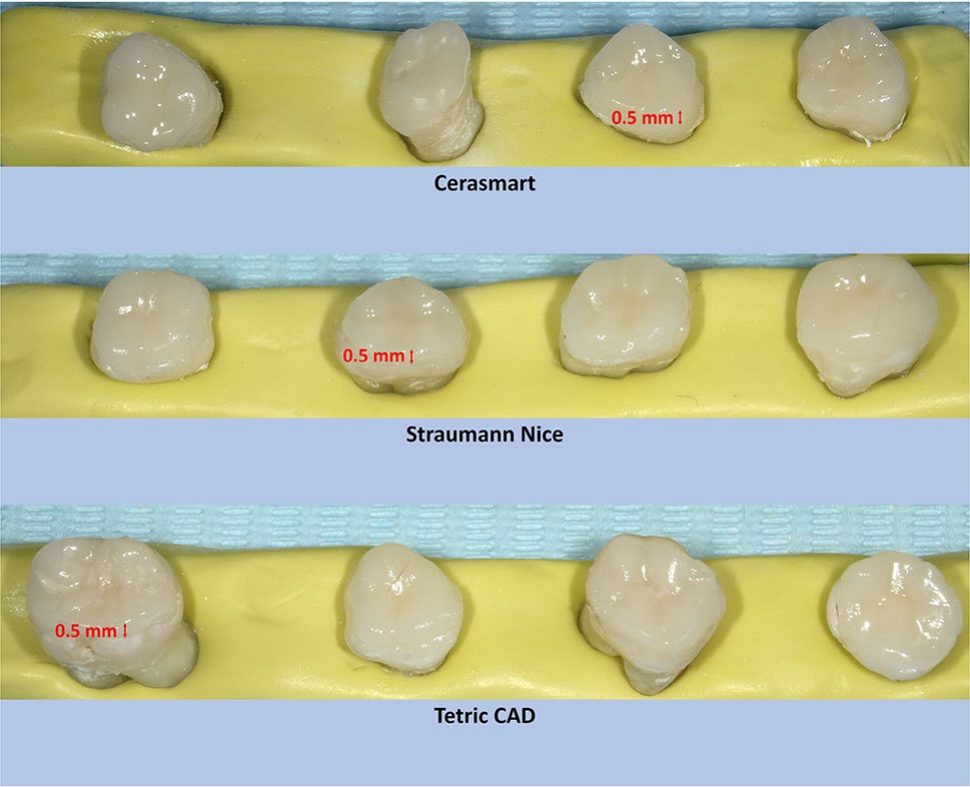
Occlusal veneer-ceramic blocks after fabrication process

Occlusal veneers were fabricated with a milling machine (PlanMill 40, Planmeca, Helsinki, Finland). The restorations were milled with a thickness of 0.5 mm (Fig. 3). After milling, the sprues were cut out using a high-speed diamond bur and the restorations were verified for imperfections and adequate fit.

### Cementation procedures

The correct adhesive cementation protocol will enhance the strength of those minimally invasive restorations. All specimens (*n* = 90) were subjected to the cementation procedures. Before the cementation technique, inner surfaces of Cerasmart and Tetric CAD occlusal veneers were air-abraded using aluminum oxide at 1.8 bar of pressure (Basic Quattro 230/240, Renfert GmbH). After sandblasting, the restorations were cleaned in an ultrasonic unit with 70% ethanol, thoroughly rinse with water spray and gently air-dried with oil-free compressed air. Regarding the Cerasmart restoration material, a silane coupling agent (Monobond S, Ivoclar Vivadent) was applied and left for 1 min, then air-dried. Regarding the Tetric CAD restoration material, a thin layer of universal bonding agent (Adhese Universal, Ivoclar Vivadent, Schaan, Liechtenstein) was applied and scrubbed for 20 s on the conditioned surfaces. After this, the Adhese Universal was dispersed with oil-free compressed air and light-cured.

The Straumann Nice restorations were etched with 5% hydrofluoric acid (Dentobond Etch, Itena, France) for 20 s, washed, dried and coated with a silane coupling agent Monobond S (Ivoclar Vivadent, Schaan, Liechtenstein). Afterwards, phosphoric acid 37.5% was used to etch all teeth (Total Etch, Ultradent, USA), for 20 s [29]. The prepared teeth were rinsed with a vigorous stream of water and dried with compressed air until the etched enamel surfaces appeared chalky white. Following that, the teeth surfaces were treated with Adhese Universal, scrubbed into the teeth surface for at least 20 s and light-cured for another 20 s.

The restorations were cemented with an adhesive dual-cure resin cement (Variolink Esthetic DC–Ivoclar Vivadent, Schaan, Liechtenstein). The marginal excess cement was removed, then each of the tooth surface (buccal, lingual, mesial, and distal) were light-cured, for 20 s. The restored teeth were stored in physiological serum at room temperature (22 ± 1 °C) prior to testing.

### Acidic artificial saliva preparation and specimen’s exposure

The acidic artificial saliva preparation protocol was performed according to the modified method of Alves and co-workers [30]. In the first phase, the artificial saliva was obtained by weighing NaCl, KCl, CaCl_2_·2H_2_O and CO(NH_2_)_2_ in mass ratio of 1:1:2:2.5. After weighing, the raw were dissolved in 1 L of distilled water, until a clear solution was obtained. The pH solution was measured using a Thermo Scientific Eutech pH 150 portable pH meter, with electrode (Thermo Scientific, Waltham, Massachusetts, USA) and the value obtained was 7.141 at 18° ± 2 °C. To obtain an acidic artificial saliva, the basic saliva was lowered to 2.939 value at 18° ± 2 °C, by adding HCl 37% [31]. Following cementation, 60 specimens (20 for each CAD/CAM material block considered), were subjected to acidic artificial saliva at 37 ± 1 °C, using an orbital stirrer incubator (ES20/60 Biosan, Riga, Latvia), for 1 month and then subjected to compressive strength test, after half of specimens (*n* = 30) were thermo-cycled before.

### Thermal cycling

Half of the specimens exposed (*n* = 30) and all the specimens non-exposed to acidic artificial saliva (*n* = 30), were subjected to thermal cycling, before fracture resistance test. The thermo-cycled study (1000 cycles) for each specimen (SD Mechatronik GmbH Thermocycler, Feldkirchen-Westerham, Germany), representing 1 month of clinical service, before compressive strength test [32]. The thermo-cycled study is based on the alternations of each specimen between 5 and 55 °C ± 2 °C, according to International Standards Organization (ISO) 11405 [33]. It was taken into account a 5 s repose transfer time between each bath [34]. At the end of thermo-cycled study, all the specimens were subjected to static fracture load test and afterwards, the teeth were carefully evaluated by scanning electron microscopy to identify the cracks or restorations fracture.

### Compressive strength test

**Fig. 4:**
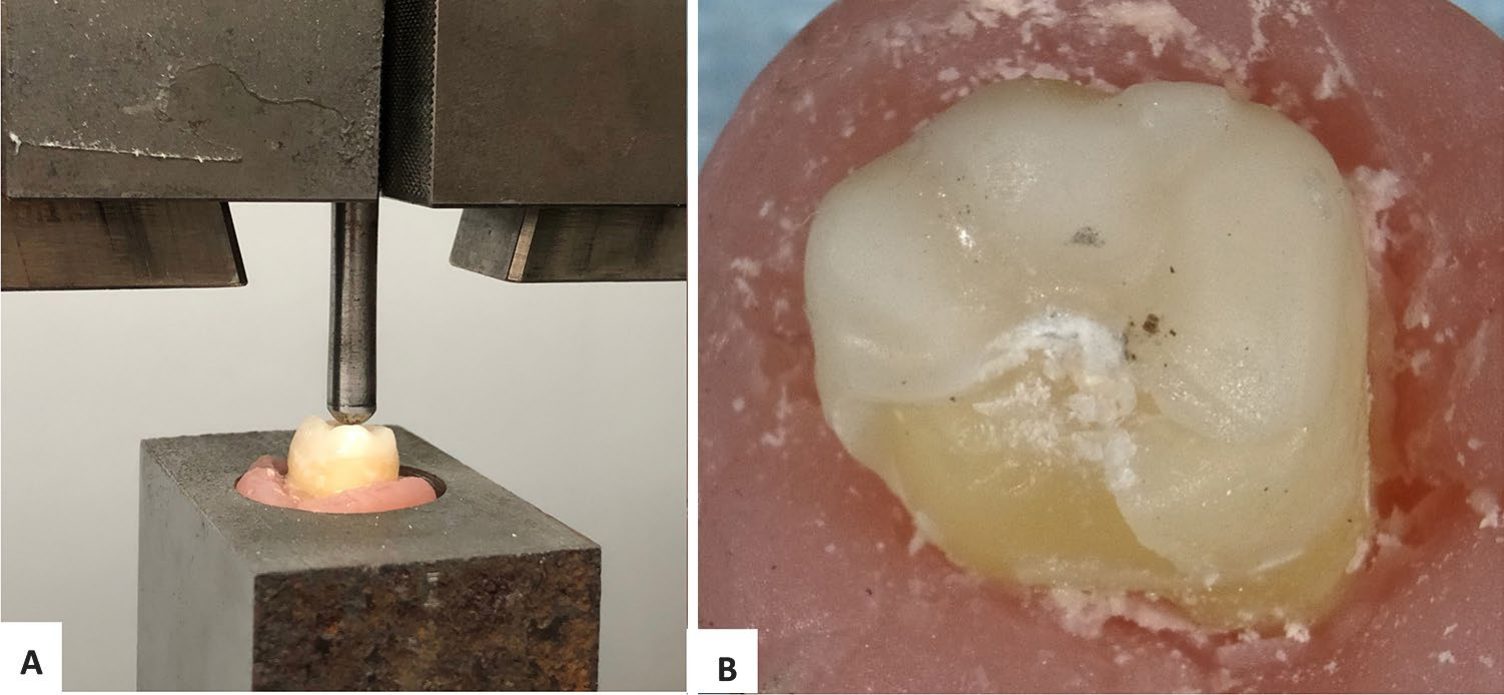
**A** Molar tooth fixed in testing machine under the long axial rod; **B** fractured occlusal veneers after the compression test, using a force of 1919 N (Straumann Nice specimen)

To determine the static fracture load, on both groups of specimens (exposed and non-exposed to acidic artificial saliva), a vertical compression test was performed, using a universal testing machine (Zwick Proline Z005, Ulm, Germany). First, the molars were inserted in an acrylic hollow cylinder made from acrylic glass. The apical part of the tooth was embedded in polyvinyl siloxane low consistency to simulate human periodontium and the resilience of teeth. Using the universal testing machine, the specimens were loaded until fracture, by applying a single static compressive maximum force of 5 kN, at a speed of 1 mm/min (Fig. 4A), given by a stainless-steel bar with hemispherical head, attached to the upper movable compartment. The testing parameters were: room temperature of 22 °C ± 1 °C, a preload force of 0.1 N (Newton) and a speed of 1 mm/min, recorded by a computer. For maximum accuracy and reproducibility, Zwick Proline Z005 machine was employed, with a high resolution of the measured value (24-bit). This machine enrolls with accuracy any minimal force changes applied on the samples. When the restorations were fractured, the compressive test was considered completed (Fig. 4B).

### Scanning electron microscopy (SEM)

After mechanical testing, when the static loading forces were applied, the degree of surface damage of the occlusal veneers of both groups of specimens (exposed and non-exposed to acidic artificial saliva), were examined using a scanning electron microscope (Inspect S, FEI, Tokyo, Japan). SEM analysis parameters were high vacuum (HV) mode, 20 kV, ETD (Everhart–Thornley detector for secondary electrons), with two magnification orders: 100× general overview for the specimens which are not exposed to acidic artificial saliva and another, at a higher surface topography (250×) for the specimens exposed to acidic artificial saliva. The failure degree of restorations surface damage, before and after immersion in acidic artificial saliva, was assess according to the following classification [14]:

- First failure degree (I)—the appearance of extensive cracks at the surface of restorations;
- Second failure degree (II)—the restorations were fractured;
- Third failure degree (III)—both restorations and tooth structure were fractured;
- Fourth failure degree (IV)—the appearance of longitudinal and profound fractures of the restorations as well as tooth structure fractures.

**Fig. 5:**
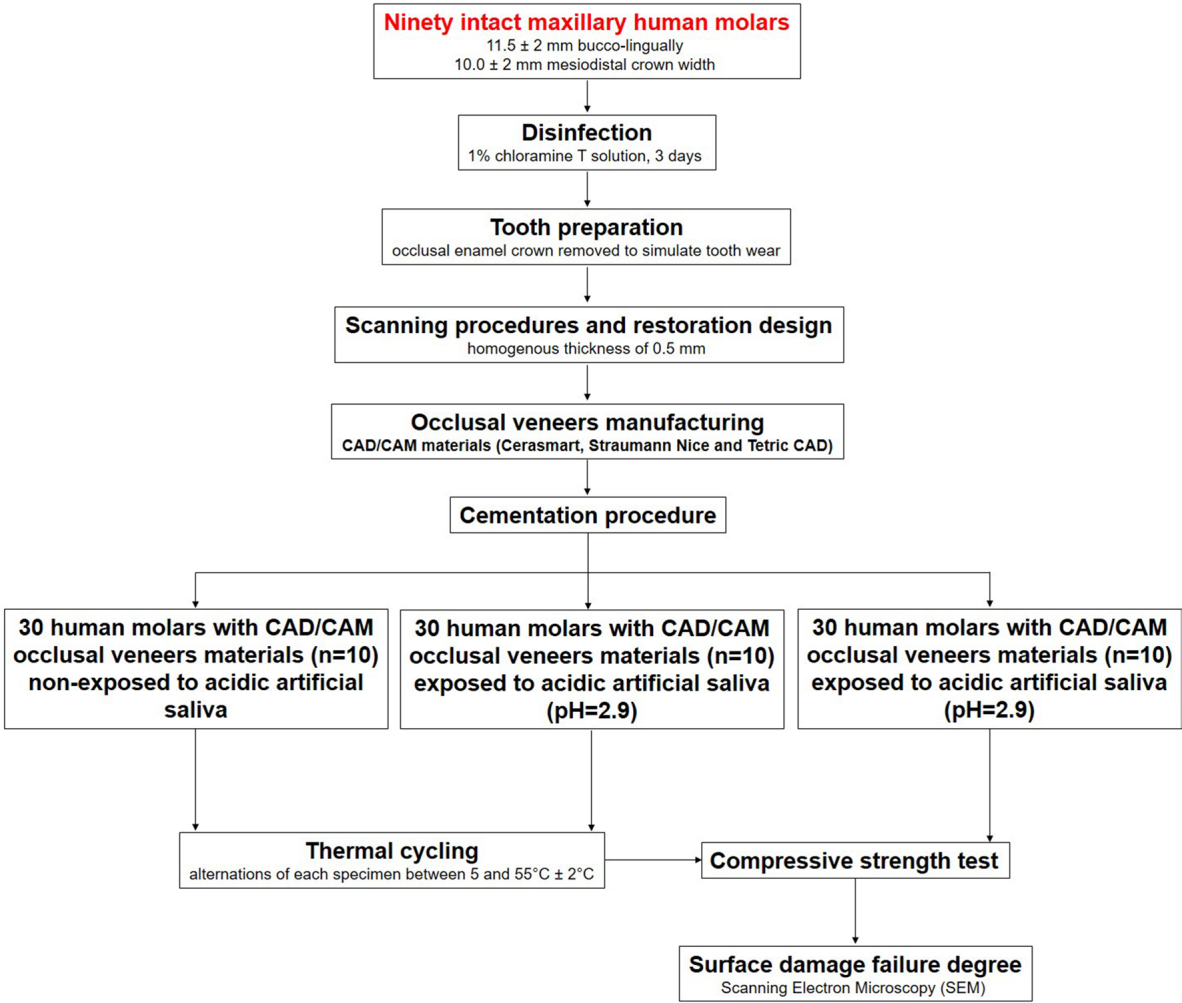
Schematic protocol of the study

For a better understanding of the experimental part effectuated in this study, in Fig. 5 is pictured the entire schematic protocol.

### Statistical analysis

Using Microsoft Excel, 2013, data were coded and tabulated. The data distribution was checked using MedCalc statistical software. The Kolmogorov–Smirnov test for normality was used to determine if the data were parametric or non-parametric. The data gathered before the acidic artificial saliva exposure were parametric; therefore, one-way ANOVA was conducted followed by Student–Newman–Keuls test for all pairwise comparisons (as a post-hoc analysis) to further assess the data. For the data gathered after the acidic artificial saliva exposure, a non-parametric equivalent (Kruskal–Wallis test) followed by Conover’s test of multiple comparisons was used to analyze the data. The *p* value of statistical significance level was set to *p* < 0.05. The started null hypothesis was that there would be no difference in fracture load resistance between the three tested materials.

## Results

### Compressive strength test

#### Non-exposed specimens to acidic artificial saliva

**Fig. 6:**
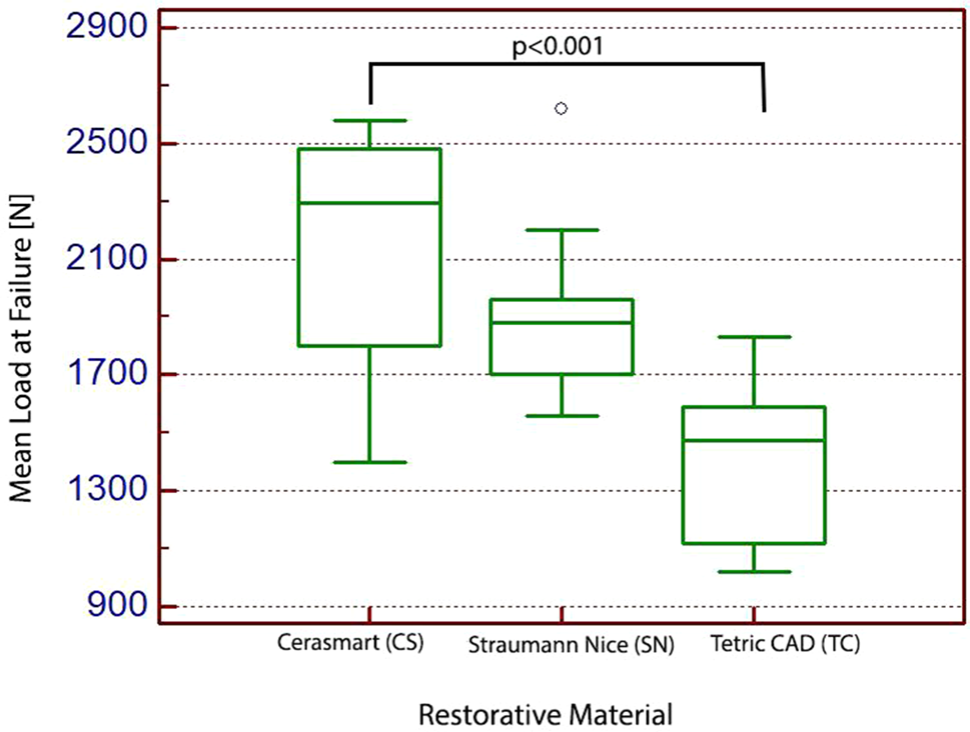
Box plots showing compressive strength mean value for the non-exposed specimens to acidic artificial saliva

After thermo-cycling study, thirty human molars with occlusal veneers of three type of CAD/CAM restorative material (Cerasmart, Straumann Nice and Tetric CAD) were subjected to compressive test. The one-way ANOVA test returned a value of *p* < 0.001, rejecting the null hypothesis and showing that the results have strong statistical relevance. The post-hoc analysis results indicate that the most resistant out of the three tested CAD/CAM restorative materials is Cerasmart with a mean value of 2131 N followed by Straumann Nice (mean value of 1919 N) and the least resistant being Tetric CAD, with a mean value of 1413 N (Fig. 6). To test the significant differences between failure types within each group, the mean load of each type of failure was calculated and statistically analyzed. Table 2 shows the compressive strength mean values and standard deviations for each group of CAD/CAM restorative materials, with Student–Newman–Keuls test post-hoc for all pairwise comparisons between groups.

**Table 2:** Means and Standard deviation [SD] values for non-exposed restorative materials to acidic artificial saliva and comparisons between means (each, *n* = 10)

CAD/CAM restorative material	Thickness [mm]	Mean [N] ± SD	Different (*p* < 0.05) from CAD/CAM restorative material number
(1) Cerasmart	0.5	2131 ± 441.2	(3)
(2) Straumann Nice	0.5	1919 ± 306.2	(3)
(3) Tetric CAD	0.5	1413 ± 276.5	(1)(2)

#### Exposed specimens to acidic artificial saliva

After 1 month of immersion in acidic artificial saliva at 37 °C, followed by a thermo-cycled test, thirty human molars covered with occlusal veneers from CAD/CAM restorative materials, were subjected to fracture test, to investigate the effect of acidic artificial saliva and the thermal cycling process on ultrathin restoration thickness of the CAD/CAM material blocks. The results of the Kruskal–Wallis test revealed that the exposure to acidic artificial saliva and thermal cycling process, significantly affected the compressive strength mean values regarding the type of the CAD/CAM restorative material used (*p* = 0.0006).

**Fig. 7:**
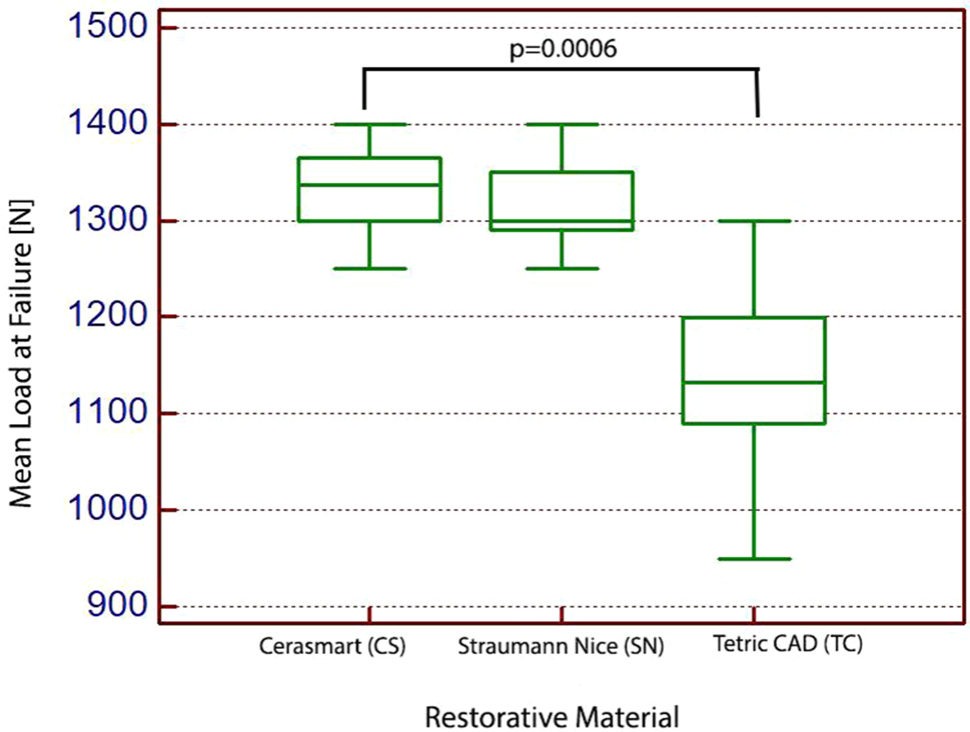
Box plots showing fracture resistance mean value for the exposed specimens to acidic artificial saliva

The Conover’s test results revealed that, after 1 month of exposure to acidic artificial saliva, the most resistant CAD/CAM restorative material is Cerasmart (mean value—1333 N), followed by Straumann Nice (mean value—1313 N) and Tetric CAD, with a mean value of 1135 N. Compressive strength mean values for all the exposed tested groups, to acidic artificial saliva, are illustrated in Fig. 7. Table 3 shows the compressive strength mean values and standard deviations for each group of CAD/CAM restorative materials, with Conover’s test for multiple comparisons between groups.

**Table 3:** Means and Standard deviation [SD] values for restorative materials exposed to acidic artificial saliva, as well as comparisons between means (each, *n* = 10)

CAD/CAM restorative material	Thickness [mm]	Mean [N] ± SD	Different (*p* < 0.05) from CAD/CAM restorative material number
(1) Cerasmart	0.5	1333 ± 44.6	(3)
(2) Straumann Nice	0.5	1313 ± 42.5	(3)
(3) Tetric CAD	0.5	1134 ± 115.4	(1)(2)

#### Exposed specimens to acidic artificial saliva without thermal cycling

**Fig. 8:**
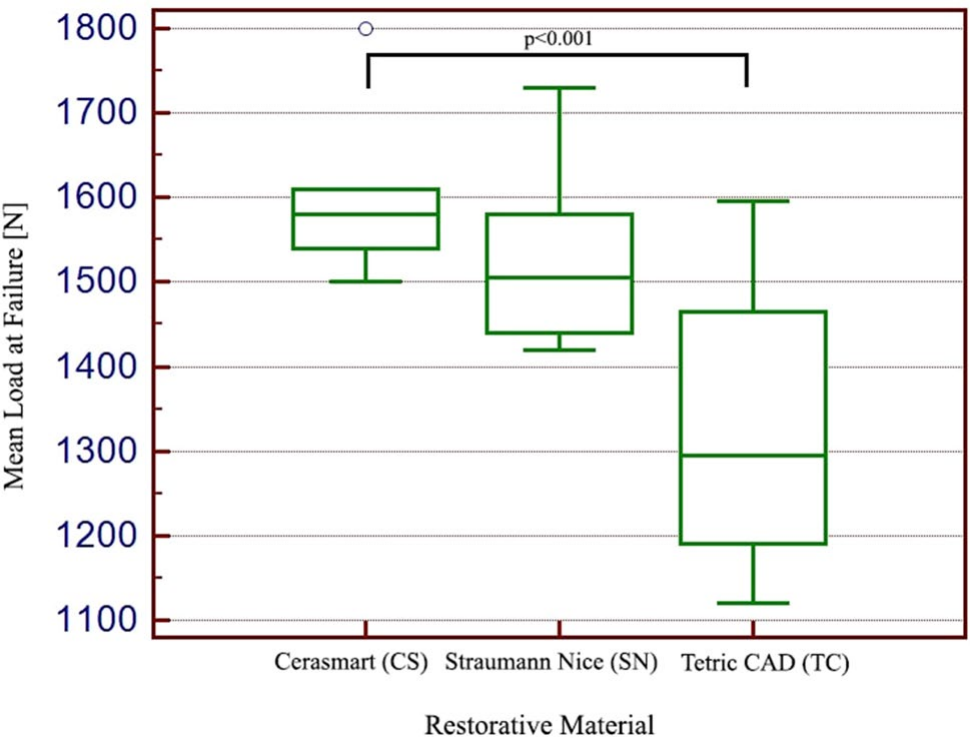
Box plots showing fracture resistance mean value for the exposed specimens to acidic artificial saliva, without thermal cycling study

To investigate only the influence of acidic artificial saliva on the three type of CAD/CAM restorative material (Cerasmart, Straumann Nice and Tetric CAD), another 30 human molars covered with occlusal veneers were subjected to compressive test, after 1 month of immersion in acidic artificial saliva at 37 °C. In Fig. 8 are illustrated the compressive strength mean values for the specimens exposed to acidic artificial saliva, without thermo cycling study.

The one-Way ANOVA test returned a *p* value of *p* < 0.001 rejecting the null hypothesis and showing that the results show statistical relevance. The results indicate that the most resistant out of the 3 tested materials is Cerasmart with a mean of 1591 N followed by Straumann Nice with a mean of 1517 N and the least resistant being Tetric CAD with a mean of 1325 N.

### SEM analysis

**Fig. 9:**
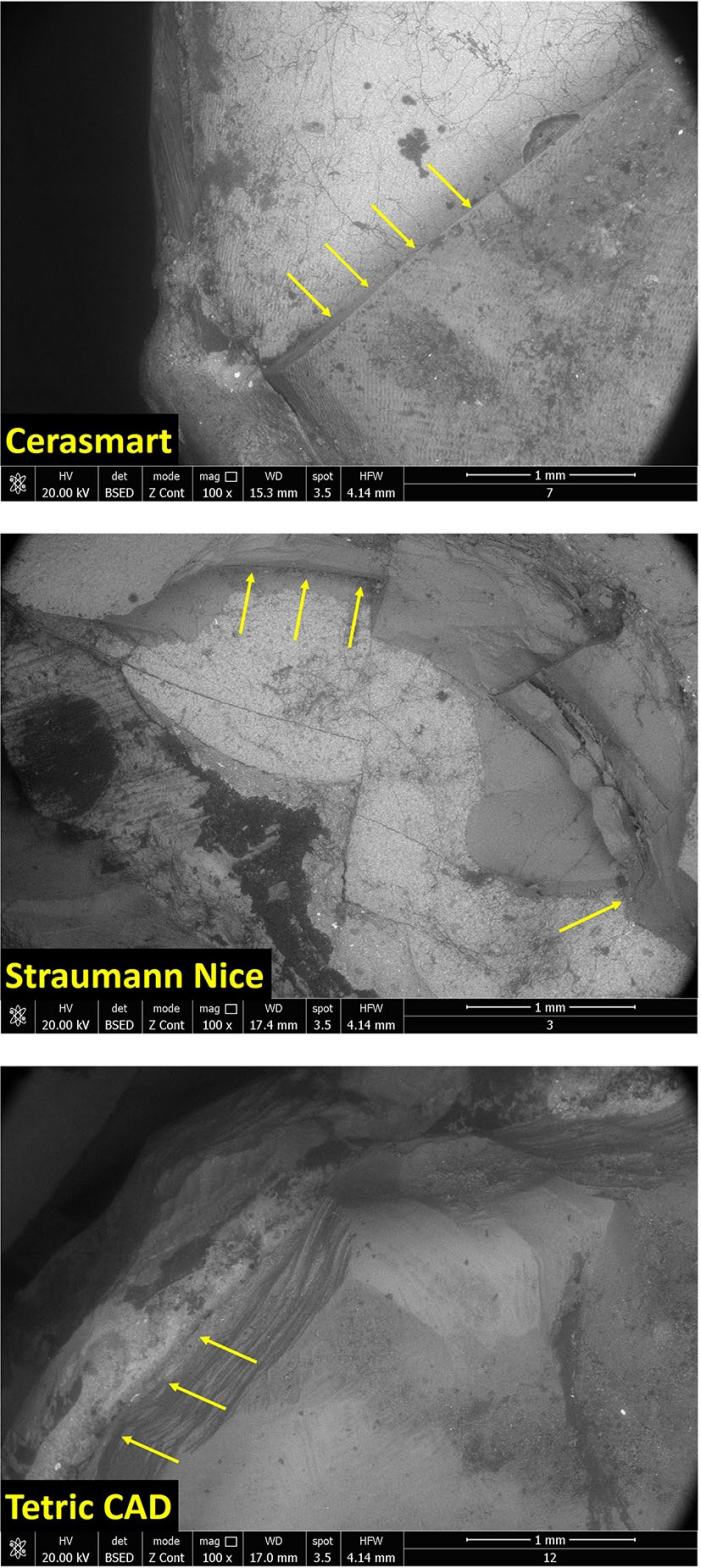
SEM images of non-exposed specimens (Cerasmart, Straumann Nice and Tetric CAD), after compressive load forces. The magnification 100× demonstrates the loading point as the site for initiation of the critical crack, at 2131 N (for Cerasmart), 1919 N (for Straumann Nice) and 1413 N (for Tetric CAD)

The occlusal veneers surface damage degree, of both groups of specimens, after the compression test, were investigated by SEM. The images of the morphology of non-exposed specimens (*n* = 30; 10 for each restorative material) to acidic artificial saliva are presented in Fig. 9. After the compression test, the restorations fractures degree was assessed by SEM examination (7 representative specimens). Changes in surface topography before exposure, were observed at the morphological evaluation conducted in the present study. Restorations fractured surfaces consisted in fine hackle lines and cracks propagation (highlighted by yellow arrows). At the contact point between each restorations and stainless-steel with hemispherical head, appear a sinuous crack lines which radiates in all directions with cohesive and adhesive displacement, some of them being in-complete due to the polymeric bridge formation.

For the specimens unexposed to acidic artificial saliva, the most common degree of surface damage of the occlusal veneers in all groups were first (I) and second (II) failure degree, meaning the appearance of extensive cracks as well as fracture involving only the restoration.

**Fig. 10:**
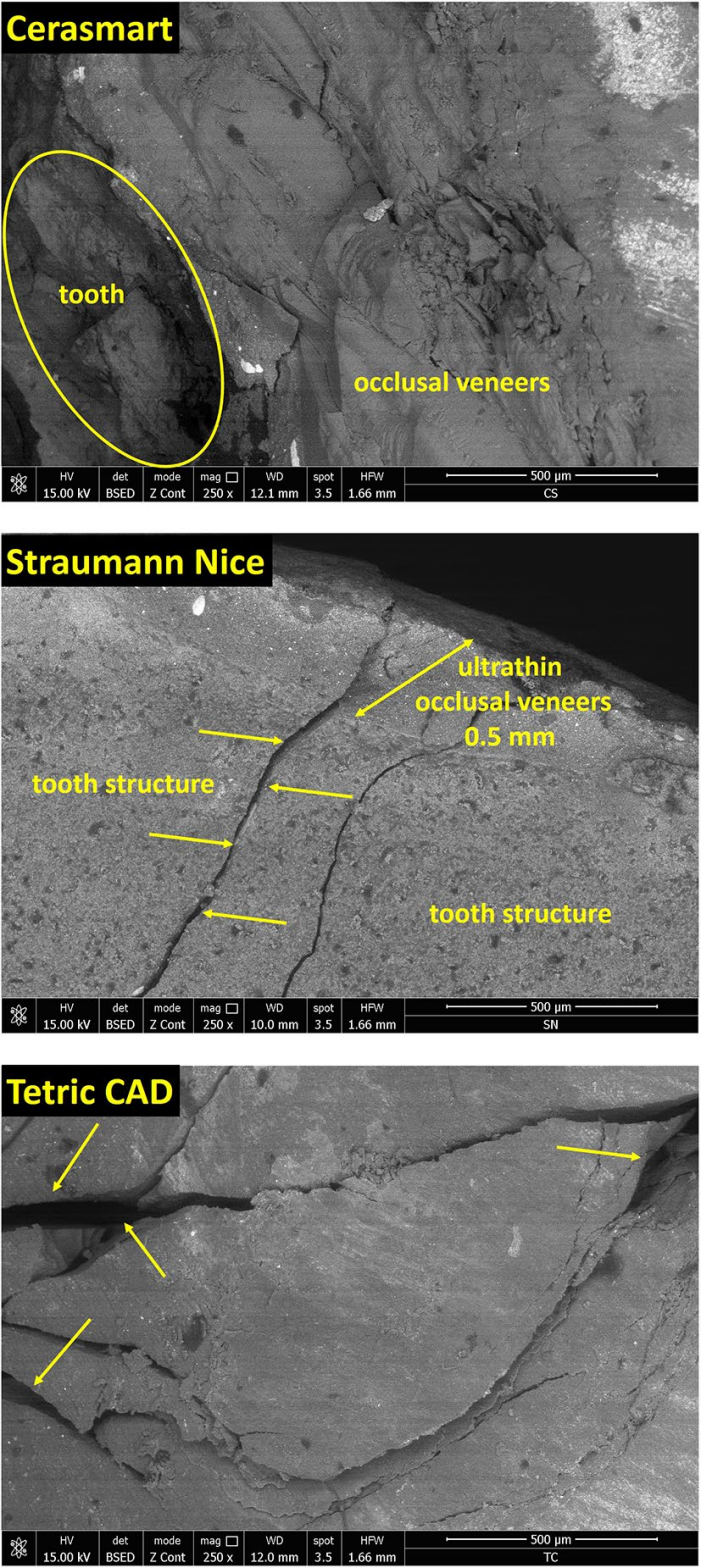
SEM images of the exposed specimens (Cerasmart, Straumann Nice and Tetric CAD), after compressive load forces. The magnification 250× demonstrates longitudinal and profound fractures of restorations and tooth structure, at 1333 N (for Cerasmart), 1313 N (for Straumann Nice) as well as extensive cracks formation at the surface of restorations and fracture involving only the restoration at 1135 N (for Tetric CAD)

In Fig. 10 are depicted the SEM images of the specimens exposed to acidic artificial saliva (*n* = 30, 10 for each restorative material). The fracture degree of the ultrathin occlusal veneers fabricated from CAD/CAM material blocks, are much higher compared to the non-exposed specimens at acidic artificial saliva. Immersion in acidic artificial saliva for 1 month had an extremely significant effect on all three types of CAD/CAM restorative materials. Under the pressure of the static loading force, the ultrathin occlusal veneers from CAD/CAM restorative materials were crushed, affecting even the tooth structure. At the contact point between the occlusal veneers surface and the stainless-steel bar with hemispherical head, appear the cracks, usually initiated from the central notch. In some cases, the chipping of the tooth, appear due to the extended fracture damage until the tooth structure (highlighted by the yellow circle). No fracture involved pulpal tissue.

The most common degree of surface damage of the occlusal veneers in Cerasmart and Straumann Nice groups were third (III) and fourth (IV) failure degree, meaning both restorations and tooth structure were fractured; as well as the appearance of longitudinal and profound fractures of the restorations and tooth structure fractures. As regarding the Tetric CAD restorative material, the most common degrees of surface damage were first (I), second (II) and third (III) failure degrees, meaning the appearance of the extensive cracks and fractures, involving both the restoration as well as the tooth structure.

## Discussion

Nowadays, the conservation of natural morphology of teeth structure is considered the most important objective in restorative dentistry. The management of tooth wear is focused on an earlier clinical detection of the changes that can occur on morphology of teeth. Tooth wear is a physiological age dependent process which can lead to the loss of enamel and dentin, cause pain and quality of life decreasing [35]. Tooth wear is considered to be analogous to bruxism—a pathology considered to be another cause of wear. Bruxism is a multifactorial etiology, which comes from the mechanical wear of the teeth, being limited to their contact surface. Bruxism is considered to be the most harmful parafunctional activity of the stomatognathic system, being responsible for tooth wear, periodontal tissue damage as well as joint and/or muscle lesions. Prosthetic restoration does not solve this pathology, due to the fact that it treats the effect and not the cause. Moreover, dental malocclusion which is considered a cause of bruxism, being a peripheral factor can be treated by prosthetic restoration [36, 37]. The relationship between bruxism and dental wear remains questionable, due to the fact that in literature are various studies which have demonstrate the positive relationship [38, 39], but other research studies refute this relationship [40, 41]. Occlusal interventions and occlusion protection devices (occlusal splints) are the most commonly treatments used in the management of bruxism. The occlusal interventions can be performed either by occlusal adjustment or by occlusal rehabilitation using composite materials [42].

The latest dental materials used to restore worn dentition through indirect techniques tend to combine flexibility and strength, both needed when minimal preparation is required and the durability of ceramic materials is influenced by their brittleness, because most of the clinical failures are related to crack propagation. The degradation of dental material based on resin composites is a crucial factor which affects the efficiency of direct and indirect restorations [43]. The degradation resistance of a dental material based on resin composites is a crucial requirement for their oral use, representing a key factor in selecting the type of restoration material. It should not be neglected the fact that tooth restorations are exposed to various complex and oral conditions for a long time. For this purpose, special attention must be paid to the surface behavior of these CAD/CAM restoration in the presence of extrinsic and intrinsic acidic substances. It is well-known that acid attacks the surface of the tooth altering his structure over time [44]. The most prone patients in this case are the individuals with gastroesophageal reflux, because they have frequent regurgitation of gastric acid from the stomach into the esophagus or oral cavity [45]. Dental restorations of these patients are daily exposed to oral acid environment, which affects their mechanical properties. Even an intake of carbohydrates leads to the production of organic acids by dental plaque and thus to the decrease of the pH in the oral cavity (around 4.5) [46]. Although dental material type ceramics are resistant to chemical attack, their composition may be affected by the medium pH, the exposure time and the temperature of the exposure [47]. Knowing how these CAD/CAM restorative materials behave in the presence of an acidic medium for a certain period, can guide the dentist in selecting the proper materials for patients with gastroesophageal reflux or other analogous diseases.

In this regard, the present study was conducted to evaluate if exposure to an acidic medium of ultrathin occlusal veneers fabricated from three types of CAD/CAM restorative materials (a nanoceramic resin—Cerasmart; a glass ceramic—Straumann Nice and a composite resin—Tetric CAD) affects their mechanical features, more precisely the fracture strength.

We started from the hypothesis that 0.5 mm ultrathin occlusal veneers will be as resistant as thicker occlusal veneers of 0.7–1 mm when applying a compressive force higher than the normal masticatory forces. According to the manufacturer’s recommendations, the ideal thickness must be between 0.5 and 1.5 mm, but the daily practice of these values may vary, that’s why, in the present study it was chosen the minimal value to evaluate if even for an ultrathin restoration there are predictable results regarding the compressive strength forces, because occlusal forces on posterior teeth are high. The three selected materials encompass the requirements for resistance and elasticity even in a thin layer of 0.5 mm. Moreover, we assumed that immersing the entire tooth-veneer complex in acidic artificial saliva followed by performing the thermocycling study, contributes to the deterioration of its structure. That is why we wanted to determine the value of the maximum compression force that can be applied to the entire complex and the degree of damage of the occlusal veneers with ultrathin dimensions (0.5 mm), both for the specimens immersed in acidic artificial saliva or subjected to thermocycling, as well as for the specimens immersed in acidic artificial saliva and then subjected to thermocycling. The assumption that acidic artificial saliva would not affect any type of restorative materials considered, was rejected, because all three restorative materials suffered changes regarding their compressive strength. To test the significant differences between failure types within each group, the mean load of each type of failure was calculated and statistically analyzed, before and after exposure to acidic artificial saliva.

First of all, it was analyzed the specimens which comprises only the human molars covered with occlusal veneers from CAD/CAM restorative materials that were subjected to thermocycling, without being exposed to acidic artificial saliva. The outcomes from the static load tests, after thermo-cycling study, exhibited a mean failure load of 2131 N for the nanoceramic resin veneers (Cerasmart), 1919 N for the glass ceramic and 1418 N for the composite resin (Tetric CAD). Our results are higher than the failure loads, or comparable with those described in the literature for similar restorations. For example, Zamzam et al. [48] investigated the load capacity of lithium disilicate restorations under lateral static loading. The author’s results exhibited a mean failure load of 493.21 ± 102.24 N. Yildiz et al. [49] reported that the mean fracture load was 2356 N regarding the restorations of 1.5 mm thickness, made from glass ceramic. Another study conducted by Clausen and co-workers [50], revealed a mean fracture load of 4070 N for restorations fabricated from glass ceramics, having notch and cusps thickness ranging between 1.5 and 2.0 mm. Sasse and co-workers [15] showed that the median fracture load was 2355 N for lithium disilicate restorations, with a notch thicknesses of 0.5 mm and cusps thickness of 0.8 mm. Al-Akhali and co-workers [51], reported that 0.8 mm lithium disilicate restorations thickness demonstrated substantially higher fracture resistance (1.545 ± 175.2 N mean failure load), but still slightly smaller than the mean failure load obtain in the present study (1919 ± 306.2 N). However, the differences between studies can be attributed either to the different load configurations used or to the differences in occlusal thickness. In the present study, the occlusal veneers restorations were milled to a precise thickness of 0.5 mm.

Second, it was analyzed the specimens covered with occlusal veneers from CAD/CAM restorative materials which have been exposed to acidic artificial saliva followed by the thermal-cycle test. After statistical analyses, the null hypothesis was rejected and the results revealed that both the exposure to acidic artificial saliva and thermal cycling process, significantly affected the compressive strength mean values regarding the type of the CAD/CAM restorative material used. The most resistant CAD/CAM restorative material is Cerasmart (mean value—1333 N), followed by Straumann Nice (mean value—1313 N) and Tetric CAD, with a mean value of 1135 N. Making a comparison between the results obtained for the first group of specimens (subjected only to thermocycling) and the second group of specimens (subjected to acidic artificial saliva and thermocycling), a mean compressive strength of 798 N (for Cerasmart), 606 N (for Straumann Nice) and 279 N (for Tetri CAD) can be observed, which is due only to the acidic artificial saliva exposure. It can be concluded that, 1 month immersion in acidic saliva, affects the compressive strength of all the considered restorations, i.e., these CAD/CAM restorative materials are less resistant to static load force than non-exposed restorative materials, however, surpassing the normal bite forces. Regarding the specimens exposed to acidic artificial saliva, these restorative materials withstood the application of a lower compression force than the force applied on the specimens which were not subjected to acidic saliva, and therefore, the acidic artificial saliva influences the CAD/CAM restorative material evaluated in the present study. Albelasy et al. [24] evaluate the effect of the restoration thickness (1 vs. 1.5 mm) of a lithium disilicate glass ceramic and a resin composite CAD/CAM materials, after immersion in artificial saliva for 6 months on the compressive strength of restorations. They found that the type and thickness of restoration material substantially affects the compressive strength values. Contrary, the samples which were immersed in artificial saliva for 6 months, had no significant effect on mean fracture resistance values. Moreover, Ioannidis et al. [52], found no substantial difference between compressive strength of 0.5 and 1.0 mm restorations thickness from resin composite. Another factor which explains the difference could be the fluctuation in the bonding substrate. The fluctuation in the bonding substrate was evaluated by Elsayed [53] when comparing the compressive strength of thin and ultrathin occlusal veneers constructed from glass ceramics and hybrid ceramics, bonded to different bonding substrates. The author found that the immediate dentin sealing concept (immediate treatment of the freshly cut dentin after exposure by the bonding agent) showed the highest mean compressive strength followed by enamel and delayed dentin sealing.

Significant difference was observed when comparing the results from both groups of specimens (exposed and non-exposed to acidic artificial saliva), on compressive strength. In the present study, a large standard deviations was obtained, which could be attributed both the natural variations regarding tooth properties and anatomy as well as errors occurred during the preparation and the CAM processing. It’s more likely that these variations exist in clinical situations; therefore, the range values can be considered relevant as it was connected to the actual performance.

Third, it was analyzed the specimens covered with occlusal veneers from CAD/CAM restorative materials which have been exposed only to acidic artificial saliva. Once again the null hypothesis was rejected showing that the results show statistical relevance. The most resistant out of the 3 tested materials is Cerasmart with a mean of 1591 N followed by Straumann Nice (1517 N) and Tetric CAD (1325 N) being the least resistant. By comparing the results obtained for the second group of specimens (subjected to acidic artificial saliva and thermocycling) and the third group of specimens (subjected only to acidic artificial saliva), one can observe a mean compressive strength of 258 N (for Cerasmart), 204 N (for Straumann Nice) and 191 N (for Tetri CAD), which is due only to the thermo-cycling test.

According to the results obtained, one can affirms that, the exposure to acidic artificial saliva led to a higher degradation of the CAD/CAM restorative material than termocycling process. By performing both processes (exposure to acidic saliva and thermocycling), the degradation of the CAD/CAM restorative material is much deeper, highlighted also by the SEM analysis, which shows the degradation of the entire tooth-veneer complex, from the surface to the depth. We believe that the acidic artificial saliva has corodate the entire tooth-veneer complex and the thermocycling process has weakened him, which is way the crack lines no longer radiated in all directions at the surface of the occlusal veneers, but radiated toward the tooth. However, all the considered CAD/CAM restorations at 0.5 mm thickness demonstrated compressive strength values which exceeded both the maximum chewing force (up to 900 N) [54], and parafunctional masticatory forces (780–1120 N) [55] in individuals, even after 1 month in acidic artificial saliva (Tables 2, 3 and 4). The results obtained in the present study could be owed to the adhesive cementation which makes a locked contact between the CAD/CAM restorative material, luting agent and dentinal substrate. A close contact between all three parameters can dissipate the applied force through the entire tooth, periodontal ligament even through the alveolar bone [56].

**Table 4:** Means and Standard deviation [SD] values for restorative materials exposed to acidic artificial saliva without thermal cycling study, as well as comparisons between means (each, *n* = 10)

CAD/CAM restorative material	Thickness [mm]	Mean [N] ± SD	Different (*p* < 0.05) from CAD/CAM restorative material number
(1) Cerasmart	0.5	1591 ± 82.1	(3)
(2) Straumann Nice	0.5	1517 ± 95.7	(3)
(3) Tetric CAD	0.5	1325 ± 163.6	(1)(2)

Following SEM examination, a compressive surface deformation was also observed. CAD/CAM restorative materials tested in the present study showed high compressive strength at the contact point. SEM images of all three types of restoration from CAD/CAM material blocks (before exposure to acidic artificial saliva—Fig. 9) revealed no undesirable damage to the tooth structure even at higher fracture strength values. The preponderant failure degree in all restorative occlusal veneers were I and II degrees, which are extensive cracks and fracture only of the restorations. Conversely, after exposure to acidic artificial saliva (Fig. 10), the predominant failure degree in all three restorative occlusal veneers were III and IV, meaning catastrophic longitudinal and profound fracture of the restorations as well as tooth structure fracture. Therefore, in this case, it is very likely that the damage types obtained after immersion in acidic saliva for 1 month, can be associated to the composition of the restorative materials. This may be due to the detachment of occlusal veneers from tooth structure as an extent fracture because of the static axial load. The susceptibility of a ceramic restoration to fracture is influenced by the modulus of elasticity, meaning, the more rigid a material is, the more it produces stress concentration in different critical areas, thus producing catastrophic fractures [57]. Similar to this research, other studies also reported that the detachment of the occlusal veneers appear because of the static axial load as an extent fracture [52, 58].

This study represents an in vitro simulation of oral conditions, using molars that reproduce tooth wear. A research study interested in the examination of stresses distribution during functional and parafunctional forces inspected the stress distribution in a maxillary molar restored with ultrathin occlusal veneers and an antagonistic mandibular molar. The author’s conclusions were that injurious effects from occlusal interferences from tensile stress were found in both occlusal and root structure. The non-working interference should be avoid by occlusal adjustments or by promoting canine guidance and contact forces in working and non-working movement cause significant tensile stress in the tooth root structure [56].

The results obtained in the present study can be considered promising and useful for clinical applications, especially regarding the patients with worn dentition. Knowing how these CAD/CAM restorative materials behave in the presence of an acidic medium for a certain period, can guide the dentist in selecting the proper materials for patients with gastroesophageal reflux or other analogous diseases.

According to the results obtain in the present study, it was listed below some opportunities that these CAD/CAM restorative materials present:

- Due to the fact that nowadays there is a wide range of patients which need tooth restoration with composite material, the use of an ultrathin thickness allows minimal invasive preparation of dental hard tissues;
- A minimally invasive preparation and restoration led to a better bond strength using adhesive technique between the restoration and the enamel (adhesion on dentin is less strong). The state-of-the-art adhesive technique will enhance the strength of thin occlusal veneers;
- The investigated occlusal veneers reported in the present study, showed high compression resistance, which exceeded the reported range of human masticatory forces; and
- The restorative CAD/CAM materials available on the market are constantly improving their characteristics.

Considering the outcomes, the limitations of the present study include:

- In the present study, the endurance of the minimally invasive restorations is affected by external factors (acidic artificial saliva and thermal cycling process);
- Albeit positive results have been obtained regarding the use of minimally invasive occlusal veneers, clinical success presumption cannot be made due to the slightly differences (inherent morphology, tooth sizes, the amount of enamel or dentin) which can lead to variations among results;
- Due to the fact that in the oral cavity there is no constant and high compressive force, further investigation regarding the thermomechanical aging using a mastication simulator are needed;
- Special attention should be paid to debonding rate of the restoration from the tooth surface, due to long-term physiological serum storage, to avoid tooth desiccation;
- The results of a research study can be clinically relevant if the physiological oral conditions were simulated and the investigations were applied with maximum caution; and
- Special attention should be paid to patients with gastrointestinal reflux, as the restorative material degrades faster in the presence of acidic saliva, so the most appropriate material should be carefully chosen.

## Conclusions

The present study aimed to evaluate the compressive strength of thin occlusal veneers of three type of CAD/CAM restorative materials (Cerasmart, Straumann Nice and Tetric CAD), before and after immersion in acidic artificial saliva, as well as investigation relied on thermal cycling process. It is well-known that tooth wear is a pathology that occurs more and more often, even in young patients, so a predictable technique to overcome this issue is mandatory. The present research study showed that all three types of CAD/CAM restorative materials (nanoceramic, glass ceramic and resin composite), with 0.5 mm thin thickness, and a correct cementation protocol, are suitable alternative for patients with tooth wear. The investigated 0.5 mm thickness occlusal veneers from CAD/CAM restorative materials showed a higher compressive load, compared to those immersed in acidic artificial saliva and/or submitted to thermal cycling process, values which exceeded both normal and parafunctional bite forces, including the specimens immersed in acidic artificial saliva. However, the SEM images showed that, the acidic artificial saliva and thermal cycling process influence the restorative materials resistance. It was obtained a failure degree of I and II in the case of the specimens which have been only thermo-cycled, meaning only extensive cracks and fracture of the restorations, while the samples which were immersed in acidic artificial saliva and submitted to the thermal cycled study, present longitudinal and profound fractures of both restorations and tooth. Anyway, in case of any CAD/CAM restorative materials presented in this study, no fracture involved pulpal tissue.

## Data Availability

The data sets generated and analyzed during the current study are available from the corresponding author on reasonable request.
