# Correction: Compressive strength evaluation of thin occlusal veneers from different CAD/CAM materials, before and after acidic saliva exposure

**DOI:** 10.1007/s10266-022-00783-9

**Published:** 2023-01-06

**Authors:** Codruța Ille, Elena-Alina Moacă, Daniel Pop, Luciana Goguță, Carmen Opriș, Ioana Ligia Pîrvulescu, Liane Avram, Andrei Faur, Anca Jivănescu

**Affiliations:** 1 Department of Prosthodontics, Faculty of Dental Medicine, “Victor Babes” University of Medicine and Pharmacy, Revolutiei Ave. 1989, No. 9, 300041 Timișoara, Romania; 2 TADERP Research Center—Advanced and Digital Techniques for Endodontic, Restorative and Prosthetic Treatment, “Victor Babeș” University of Medicine and Pharmacy, Revolutiei Ave. 1989, No. 9, 300041 Timişoara, Romania; 3 Department of Toxicology and Drug Industry, Faculty of Pharmacy, “Victor Babeș” University of Medicine and Pharmacy Timisoara, 2nd Eftimie Murgu Square, 300041 Timisoara, Romania; 4 Research Centre for Pharmaco-Toxicological Evaluation, “Victor Babeș” University of Medicine and Pharmacy, 2nd Eftimie Murgu Square, 300041 Timișoara, Romania; 5 Department for Materials and Manufacturing Engineering, Faculty of Mechanics, Politechnic University of Timisoara, Mihai Viteazu Ave., No. 1, 300222 Timisoara, Romania

**Correction: Odontology** 10.1007/s10266-022-00741-5

In the original publication of the article, the author “Daniel Pop” was published as co-corresponding author and co-corresponding authorship is removed from “Daniel Pop” in this article.

The original article has been updated.

